# Impact of Optimal Medical Therapy on Reintervention and Survival Rates after Endovascular Infrapopliteal Revascularization

**DOI:** 10.3390/jcm12155146

**Published:** 2023-08-06

**Authors:** Tim Wittig, Toni Pflug, Andrej Schmidt, Dierk Scheinert, Sabine Steiner

**Affiliations:** 1 Department of Angiology, University Hospital Leipzig, 04103 Leipzig, Germany; tim.wittig@medizin.uni-leipzig.de (T.W.); andrej.schmidt@medizin.uni-leipzig.de (A.S.); dierk.scheinert@medizin.uni-leipzig.de (D.S.); 2 Helmholtz Institute for Metabolic, Obesity and Vascular Research (HI-MAG) of the Helmholtz Center Munich, University of Leipzig and University Hospital Leipzig, 04103 Leipzig, Germany; 3 Department of Vascular Surgery, Sana Klinikum Borna, 04552 Borna, Germany; toni.pflug@sana.de

**Keywords:** peripheral arterial disease, chronic limb-threatening ischemia, limb salvage, major amputation, pharmacotherapy

## Abstract

Within this single-center cohort study, we investigated the impact of optimal medical therapy on all-cause mortality, major amputation-free survival and clinically driven target lesion revascularization (CD TLR) in 552 patients with peripheral arterial disease (PAD) undergoing endovascular infrapopliteal revascularization. From the overall cohort, 145 patients were treated for intermittent claudication (IC) and 407 were treated for critical limb ischemia (CLI). Optimal medical therapy (OMT) was defined as the presence of at least one antiplatelet agent, statin and ACE inhibitor or AT-2 antagonist based on guideline recommendations. About half (55.5%) of all patients were prescribed OMT at discharge, with a higher proportion in claudicants (62.1%) versus CLI patients (53.2%). Over three years of follow-up, survival was significantly better in patients with IC (80.6 ± 3.8% vs. 59.9 ± 2.9%; *p* < 0.001). There was a signal towards better survival in those patients receiving OMT (log-rank *p* = 0.09). Similarly, amputation-free survival (AFS) was significantly better in patients with IC (*p* = 0.004) and also in patients receiving OMT (78.8 ± 3.6%) compared to that in those without OMT (71.5 ± 4.2%; *p* = 0.046). Freedom from CD TLR within three years was significantly better in the IC group (*p* = 0.002), but there were no statistically significant differences for CD TLR dependent on the presence of OMT (*p* = 0.79). In conclusion, there is still an important underuse of OMT in patients undergoing infrapopliteal interventions, which is even more pronounced in CLI despite a signal for its benefit regarding all-cause mortality and major amputation-free survival.

## 1. Introduction

PAD of the lower extremities affects an estimated 27 million adults in Europe and North America and over 200 million people worldwide [1]. While only about one in five patients with PAD shows clinical symptoms in the affected extremity, all patients have a significantly increased cardiovascular morbidity and mortality [2]. It is therefore essential to rigorously treat and control cardiovascular risk factors in all PAD patients. Due to demographic trends and the increasing number of diabetic patients, more and more patients present with critical limb ischemia (CLI) and often complex, multi-vessel disease of the arteries below the knee requiring timely revascularization [3]. CLI is the most advanced form of PAD and is characterized by ischemic rest pain, gangrene and non-healing ulcers. Due to technical advances as well as new devices in the field of endovascular therapy in recent years, the use of endovascular techniques for revascularization is now widespread and has replaced bypass surgery as the treatment of choice [4]. Although the immediate success rate of below-the-knee interventions has improved significantly with new technologies and devices [5], the reintervention rate caused by restenosis remains the most important limitation to long-term success [6].

Current guidelines clearly recommend the use of antihypertensive, lipid-lowering and antithrombotic drugs as optimal medical therapy (OMT) to improve outcomes across the full spectrum of PAD patients including asymptomatic patients, claudicants and CLI patients [2,7]. In diabetic patients, the optimal control of blood glucose levels should additionally be achieved according to international recommendations. However, despite the clear evidence of benefit, optimal medical therapy is often poorly implemented, which also has been documented in prior research in the field [8,9,10].

So far, limited data also exist on the impact of OMT on reintervention and survival rates after endovascular infrapopliteal revascularization, especially in CLI patients. Two prior studies showed a benefit of statin therapy on overall survival in CLI patients after 1 year of follow-up [11,12]. In this cohort study, we aimed to investigate the prescription rate of optimal medical therapy, defined as the use of at least one antiplatelet agent, statin and ACE inhibitor or AT-2 antagonist and its impact on all-cause mortality, major amputation-free survival and freedom from clinically driven target lesion revascularization (CD TLR) in patients undergoing infrapopliteal interventions.

## 2. Material and Methods

### 2.1. Study Design and Patient Population

Within this retrospective single-center cohort study, 552 patients with symptomatic PAD (Rutherford clinical stage 2–6) were included between September 1st 2014 and December 31st 2017, who underwent infrapopliteal endovascular intervention at the Department of Angiology, Leipzig University Hospital, Germany. In case of repeated interventions (e.g., in both limbs), each patient was included only once for the first intervention during the study period. The key characteristics of this patient population have been described in a previous study focusing on the impact of drug-coated balloon angioplasty in this patient cohort [13]. The inclusion criteria were age > 18 years, symptomatic PAD defined by categories 2–6 according to Rutherford classification and a target lesion below the tibial plateau. Before referral for vascular interventions, claudicants underwent conservative treatment for at least three months, including a recommendation to participate in a supervised exercise training program. In claudicants, indications for infrapopliteal interventions were only given when patients had an unacceptably high, lifestyle-limiting disease burden and all recommended conservative therapy measures in line with current guidelines remained unsuccessful. In patients with femoropopliteal inflow interventions, additional infrapopliteal interventions were performed at the operators’ discretion in order to improve outflow and prevent re-occlusion in selected cases.

The exclusion criterion was a non-performed percutaneous transluminal intervention. The patients were classified according to clinical presentation (Rutherford classification categories 2–3 representing the intermittent claudication (IC) group and categories 4–6 corresponding to the CLI group). Patient characteristics, including pre-interventional ankle-brachial index (ABI) measurements and medical history, were obtained as part of the clinical routine at admission. Relevant medications/medication groups including antiplatelet agents (e.g., aspirin, clopidogrel), anticoagulants, statins, alternative blood lipid-lowering medications, ß-blockers, ACE inhibitors, angiotensin II antagonists and other antihypertensive drugs were recorded at admission and discharge. As recommended by current guidelines, the patients received at least one antiplatelet drug. Dual antiplatelet therapy (DAPT) was usually prescribed in cases of treatment with drug-eluting technologies. The duration of DAPT was at the discretion of the operators and depended on the location and complexity of the lesion as well as the stent implantation. In patients with an indication for oral anticoagulation (mostly due to atrial fibrillation), the antithrombotic regimen was adjusted, taking into account the type of treatment and the existing recommendations of the German Society for Angiology and ESC guidelines [14]. All patients received a recommendation of strict nicotine abstinence and participation in structured exercise training in the case of claudicants as part of their discharge letter and discharge consultation. The Institutional Review Board of the University of Leipzig approved the analysis of this dataset (EK Votum 101/23-ek) obtained from a prospectively maintained PAD database.

### 2.2. Lesion and Procedural Characteristics

Detailed information on lesion and procedural characteristics was obtained from the intervention report and review of angiograms. All treatment decisions were at the operators’ discretion. Vessel calcification was categorized as none, mild, moderate and severe based on visual estimates. Inflow vessels were defined as arteries above the tibial plateau and outflow vessels were defined as arteries distal to the ankle fork. Lesion characteristics were classified into de novo, restenotic without prior stenting and in-stent-restenosis. If residual stenosis was below 50% in the final angiogram, the procedure was considered successful. Periprocedural complications were also noted.

### 2.3. Study Definitions and Endpoints

Optimal medical treatment was defined as the use of at least one antiplatelet agent, statin and ACE inhibitor or AT-2 antagonist at discharge. Follow-up information including rates of all-cause mortality, amputation-free survival and CD TLR was retrieved by a chart review as well as census registry queries for verification of the living status up to three years after the index procedure. Major amputations were defined as any amputation above the ankle and CD TLR was defined as any repeat intervention of the target lesion due to the deterioration of clinical symptoms (i.e., increase in one Rutherford class or more, delayed or worsening wound healing, new or recurrent wound or recurrence of ischemic rest pain).

### 2.4. Statistical Analysis

All data were obtained from a prospectively maintained PAD patients database within our vascular center. For descriptive statistics, the data are presented as the number (percentage) for categorical data and the mean (+/− standard deviation) for continuous data. Differences between groups were performed using Student’s *t*-test for continuous variables or Fisher’s exact test for categorical variables, as appropriate.

Kaplan–Meier time-to-event analyses were performed to assess all-cause mortality, amputation-free survival and CD TLR over three years. Differences in the survival curves between the groups (i.e., IC versus CLI and OMT versus no OMT) were tested with the log-rank statistics. Multivariable Cox proportional hazards regression analyses were performed, including the following covariates: age, sex (female/male), body mass index (BMI), clinical status (IC/CLI), diabetes mellitus, arterial hypertension, hyperlipidemia, renal failure, coronary heart disease (CHD), heart failure, smoking status (current/previous/never), history of prior intervention, lesion length, lesion severity (occlusions/stenosis), lesion type (de novo/restenotic/in-stent restenotic), calcification (none–mild/moderate–severe), inflow intervention, outflow intervention, OMT prescription and Beta blocker use. A stepwise procedure was conducted for the variable selection, with a chosen significance level for entry of 0.15 and a chosen significance level for stay of 0.2. All analyses were performed using SPSS version 27.0 (IBM, Armonk, NY, USA), and a *p*-value < 0.05 was considered statistically significant.

## 3. Results

### 3.1. Patient Characteristics

Over the study period, 552 patients were identified as undergoing endovascular infrapopliteal interventions, and detailed patient characteristics are given in Table 1.

The included patients had a mean age of 72.8 ± 11.0 years, and approximately three of four patients were male. Most patients exhibited a high cardiovascular risk profile, with high rates of hyperlipidemia (78.9%), hypertension (95.8%) and diabetes (59.8%). Over 70% of patients suffered from CLI, as 15.6% of patients presented with ischemic rest pain and 58.2% presented with tissue loss. Patients with CLI had a significantly lower ABI on admission than those with IC (0.51 ± 0.36 vs. 0.63 ± 0.28; *p* < 0.001). There were also some differences between the groups in terms of prior revascularization procedures. While no significant differences were found with regard to any prior target limb revascularization, more CLI patients had previous vascular surgery compared with the IC group (*p* = 0.03). Furthermore, CLI patients also exhibited higher rates of co-morbidities including CHD and chronic kidney failure.

### 3.2. Lesion and Procedural Characteristics

Detailed lesion and procedural characteristics are given in Table 2. Lesions were newly detected in 416 patients (75.4%), 111 patients (20.1%) had restenosis and 25 patients (4.5%) had in-stent restenotic lesions. The majority of patients (65.8%) were intervened for total occlusions. The lesion length was, on average, 208 ± 127 mm. In 82 patients (14.9%), additional retrograde puncture from distal was required for successful lesion crossing. The simultaneous intervention of inflow vessels was performed in 229 patients (41.5%), while the simultaneous intervention of outflow vessels was performed in 10.3%. Infrapopliteal stent implantation was necessary in 117 patients (21.2%). Procedural success, corresponding to residual stenosis below 50%, was documented in 524 patients (94.9%).

Detailed differences for the IC vs. CLI subgroup analysis can be found in Table 2. Lesions were significantly longer in patients with CLI (218 ± 124 mm) than in patients with IC (179 ± 130 mm; *p* < 0.001). No relevant differences were noted between the groups with regard to the degree of calcification of the lesions (*p* = 0.93). While more stenotic lesions were found in the IC group with 62 patients (42.8%) than in the CLI group with 127 patients (31.2%), more occlusions were seen in the CLI group with 280 patients (68.8%) than in the IC group with 83 patients (57.2%). There were no significant differences in the procedural success and complication rates between the two groups.

### 3.3. Discharge Medication and Optimal Medical Therapy

Discharge medication was documented in 551 patients. In one patient, the discharge medication was unknown due to transfer to another hospital. Data for the entire study cohort and subgroups are summarized in Table 1. A total of 406 patients (73.7%) of the study population received at least both an antiplatelet agent and statin, including 107 patients (73.8%) with IC and 299 patients (73.6%) with CLI (*p* = 0.97). Optimal medical therapy consisting of at least one antiplatelet agent, statin and ACE inhibitor or AT-2 antagonist was given to a total of 306/551 patients (55.5%). Of these, 90/145 IC patients (62.1%) and 216/406 CLI patients (53.2%) received OMT (*p* = 0.080). The data for patients with and without OMT are summarized in Table 3.

Patients with OMT had a higher rate of prior target limb interventions and were more likely to have arterial hypertension, hyperlipidemia, diabetes and CHD as concomitant diagnoses. In addition, they never smoked or smoked less frequently. In contrast, the presence of OMT was significantly lower in patients with end-stage renal kidney disease (*p* < 0.001), but no differences were found in patients with chronic renal insufficiency.

## 4. Study Outcomes over 3 Years

### 4.1. All-Cause Mortality

The Kaplan–Meier estimates (±standard error) of the overall survival of the study cohort were 84.0 ± 1.6%, 76.8 ± 1.9% and 65.6 ± 2.4% at one, two and three years, respectively.

**Figure 1:**
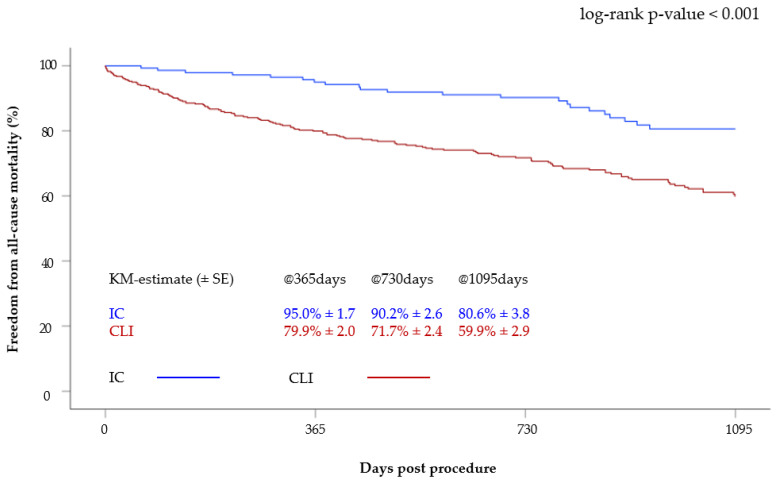
Kaplan–Meier curve of survival after 3 years according to clinical status (CLI versus IC).

Survival curves differed significantly between patients with CLI and IC (Figure 1). The survival of patients with IC was 95.0 ± 1.7% at 1 year, 90.2 ± 2.6% at 2 years and 80.6 ± 3.8% at 3 years. In the CLI group, survival at one, two and three years was 79.9 ± 2.0%, 71.7 ± 2.4% and 59.9 ± 2.9%, respectively. Thus, survival was significantly better in patients with IC (*p* < 0.001) (Figure 1).

### 4.2. Major Amputation-Free Survival

The Kaplan–Meier estimates (±standard error) of the major amputation-free survival (AFS) of the study cohort were 86.3 ± 1.8%, 80.0 ± 2.3% and 75.5 ± 2.7% at one, two and three years, respectively.

**Figure 2:**
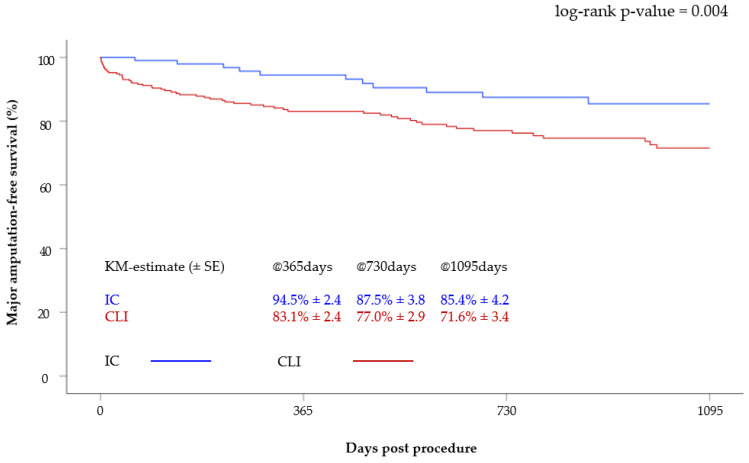
Kaplan–Meier curve of major amputation-free survival after 3 years according to clinical status (CLI versus IC).

The AFS of the claudicants was 94.5 ± 2.4%, 87.5 ± 3.8% and 85.4 ± 4.2% at one, two and three years, respectively. In the CLI group, the AFS at one, two and three years was 83.1 ± 2.4%, 77.0 ± 2.9% and 71.6 ± 3.4%, respectively. Thus, the major amputation-free survival was significantly better in patients with IC (*p* = 0.004) (Figure 2).

### 4.3. Freedom from CD TLR

The Kaplan–Meier estimates (±standard error) of the CD TLR of the study cohort was 74.8 ± 2.1%, 69.1 ± 2.4% and 65.9 ± 2.6% at one, two and three years, respectively.

**Figure 3:**
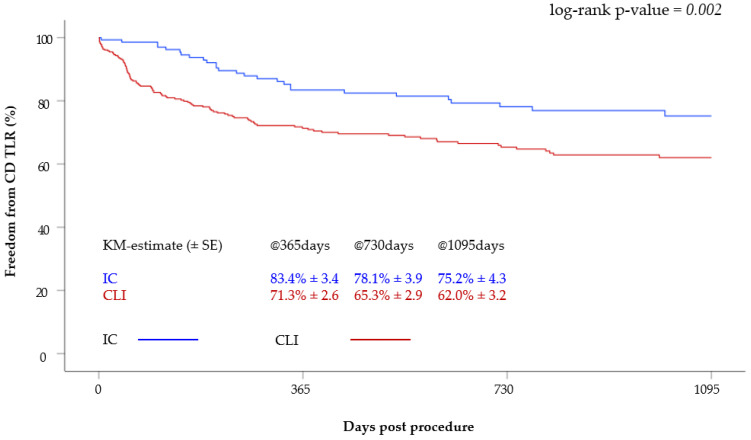
Kaplan–Meier curve of freedom from CD TLR after 3 years according to clinical status (CLI versus IC).

The freedom from CD TLR of the claudicants was 83.4 ± 3.4%, 78.1 ± 3.9% and 75.2 ± 4.3% at one, two and three years, respectively. In the CLI group, the freedom from CD TLR at one, two and three years was 71.3 ± 2.6%, 65.3 ± 2.9% and 62.0 ± 3.2%. Thus, the freedom from CD TLR within three years was significantly better in the IC group (*p* = 0.002) (Figure 3).

### 4.4. Impact of OMT on Clinical Outcomes

**Figure 4:**
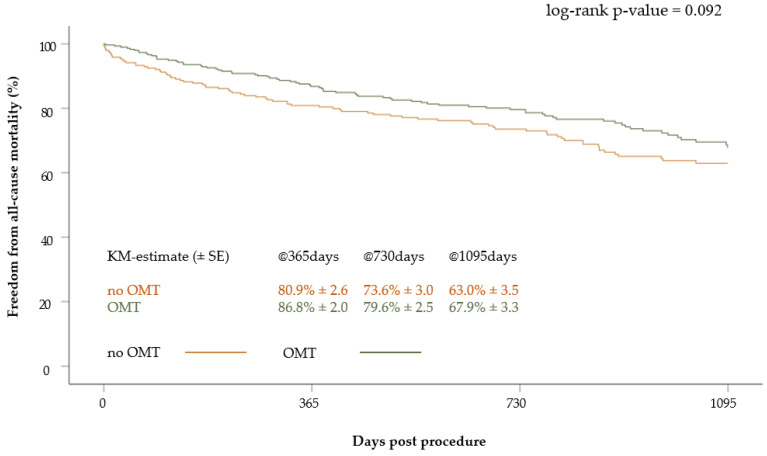
Kaplan–Meier curve of survival after 3 years according to medication at discharge (OMT versus no OMT).

The survival of patients who received OMT at discharge was 86.8 ± 2.0% at one year, 79.6 ± 2.5% at two years and 67.9 ± 3.3% at three years, respectively. In patients without OMT, the survival at one, two and three years was 80.9 ± 2.6%, 73.6 ± 3.0% and 63.0 ± 3.5%, respectively. There was a signal towards better survival in patients with OMT (*p* = 0.09) (Figure 4).

In the multivariate Cox regression analysis, the independent predictors of all-cause mortality included age (Hazard ratio (HR): 1.06, 95% confidence interval (CI) 1.04–1.08; *p* < 0.001), the presence of CLI (HR: 1.70, 95% CI 1.06–2.72; *p* = 0.03), heart failure (HR: 2.16, 95% CI 1.51–3.10; *p* < 0.001), coronary heart disease (HR: 1.50, 95% CI 1.05–2.15; *p* = 0.03), diabetes (HR: 1.46, 95% CI 1.01–2.12; *p* < 0.05) and outflow intervention (HR: 1.64, 95% CI 1.02–2.65; *p* < 0.05). Regarding pharmacotherapy, the signal of a benefit for OMT persisted (HR: 0.75, 95% CI 0.54–1.04; *p* = 0.09), with a risk reduction of approximately 25%. Interestingly, a significant mortality reduction was seen with the Beta blocker intake (HR: 0.67, 95% CI 0.47–0.94; *p* = 0.02) in the multivariate analysis.

**Figure 5:**
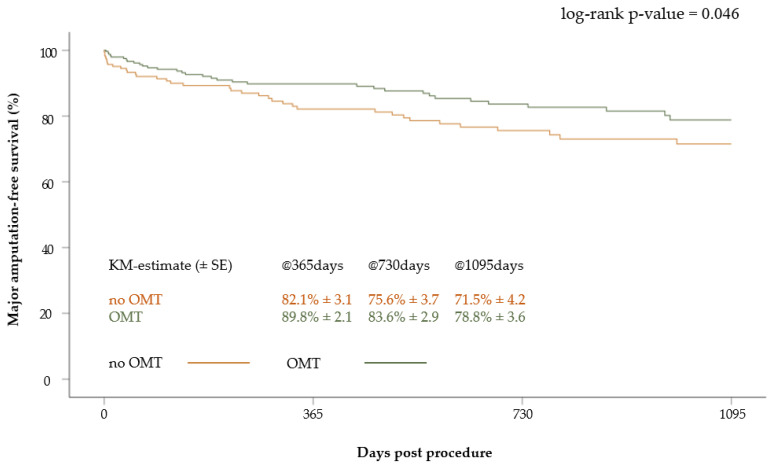
Kaplan–Meier curve of major amputation-free survival after 3 years according to medication at discharge (OMT versus no OMT).

The major amputation-free survival of patients who received OMT was 89.8 ± 2.1% at one year, 83.6 ± 2.9% at two years and 78.8 ± 3.6% at three years. In patients without OMT, the major amputation-free survival at one, two and three years was 82.1 ± 3.1%, 75.6 ± 3.7% and 71.5 ± 4.2%, respectively. Accordingly, the major amputation-free survival was significantly better in patients with OMT than in patients without OMT (*p* = 0.046) (Figure 5).

In the multivariate Cox regression analysis, the independent predictors of the combined endpoint death and major amputations included age (Hazard ratio (HR): 1.03, 95% confidence interval (CI) 1.00–1.06; *p* = 0.03), heart failure (HR: 2.36, 95% CI 1.39–4.02; *p* < 0.01), end-stage renal disease (HR: 2.54, 95% CI 1.28–5.05; *p* < 0.01) and lesion length (HR: 1.002, 95% CI 1.00–1.004; *p* = 0.02). A signal for a worse outcome was seen for the presence of CLI (HR: 1.83, 95% CI 0.91–3.70; *p* = 0.09), coronary heart disease (HR: 1.69, 95% CI 0.98–2.90; *p* = 0.06) and concomitant treatment of an inflow lesion (HR: 1.63, 95% CI 0.98–2.71; *p* = 0.06). Regarding pharmacotherapy, a weak signal of a benefit for OMT was observed (HR: 0.66, 95% CI 0.39–1.12; *p* = 0.12).

**Figure 6:**
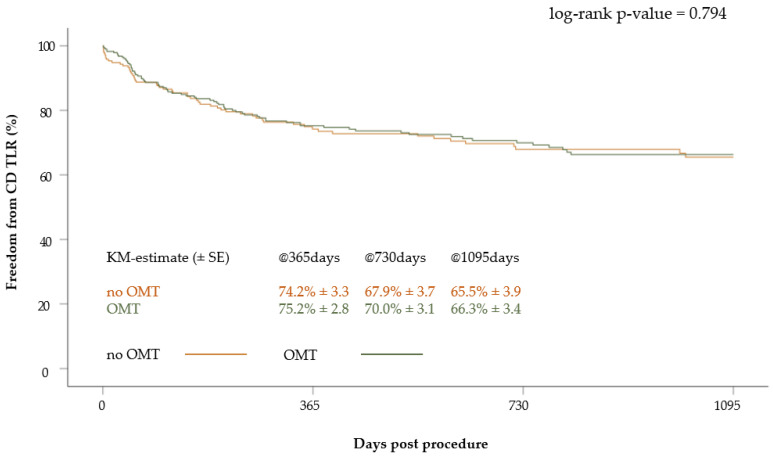
Kaplan–Meier curve of freedom from CD TLR after 3 years according to medication at discharge (OMT versus no OMT).

The freedom from CD TLR was 75.2 ± 2.8% at 1 year, 70.0 ± 3.1% at 2 years and 66.3 ± 3.4% at 3 years in patients with OMT. In patients without OMT, it was 74.2 ± 3.3%, 67.9 ± 3.7% and 65.5 ± 3.9% at one, two and three years, respectively. There were no statistically significant differences between the groups (*p* = 0.79) (Figure 6).

In the multivariate Cox regression analysis, the independent predictors of CD TLR included the presence of CLI (HR: 1.64, 95% CI 1.07–2.53; *p* = 0.02) and the treatment of occluded lesions (HR: 2.49, 95% CI 1.56–3.97; *p* < 0.01). No role of OMT was seen for this outcome.

## 5. Discussion

Current guidelines clearly recommend the establishment of optimal medical therapy with the use of antihypertensive, lipid-lowering and antithrombotic medications to improve outcomes for the full spectrum of patients with PAD, including asymptomatic patients, claudicants and CLI patients [2,7]. In diabetic patients, optimal glycemic control should also be achieved, according to recommendations. However, OMT is currently poorly implemented. In this cohort study, we examined the prescription rate of OMT, defined as the use of at least one antiplatelet agent, statin and ACE inhibitor or AT-2 antagonist and its impact on all-cause mortality, amputation-free survival and freedom from CD TLR in patients with PAD undergoing infrapopliteal intervention. Over three years of follow-up, there was a signal for both lower all-cause mortality and better major amputation-free survival in patients receiving OMT, and this finding was corroborated by Cox regression analysis after adjustment for relevant covariates. In contrast, no effect of OMT was seen for freedom from CD TLR over this time interval. The main predictors of restenosis were markers of lesion complexity such as the lesion length and the presence of total occlusions at the index procedure. In addition, the results for the subgroups of patients with IC or CLI were superior for all endpoints in patients with claudication, highlighting the detrimental prognosis of CLI.

A previous study evaluated more than 12,000 patients who received lower extremity peripheral vascular intervention for the prescription of guideline-directed pharmacotherapy, which was also defined in this study as taking an antiplatelet agent, statin and ACE inhibitor or angiotensin receptor blocker after lower extremity endovascular intervention. Only 47.4% of patients treated received the recommended medication at discharge, which means that the prescription rate in our small study cohort was slightly higher (306/551 patients; 55.5%). Interestingly, the authors also found that women and patients at the highest risk for atherothrombosis and limb loss were the least likely to be prescribed guideline-directed pharmacotherapy, underlining the need for more awareness [8]. These results were confirmed by another cohort study evaluating data from the second-largest health insurance company in Germany, BARMER, regarding patients with an index admission for symptomatic PAD. A total of 83,867 patients (average age 71.9 years and 45.8% women) were included in the study. The authors found that although women are older and have more severe symptoms at index admission for PAD, the prescription prevalence of guideline-directed pharmacotherapy, also defined as in our study, is lower in women than in men, particularly with respect to lipid-lowering agents [9]. The impact of recommend pharmacotherapy on all-cause mortality, major amputation-free survival and freedom from clinically driven target lesion revascularization (CD TLR) was not investigated in both studies [8,9]. In a nationwide study in Denmark, Subherwal et al. showed that, despite an increased use of cardioprotective medications after the incident diagnosis of PAD, the use remains modest. Throughout 18 months of follow-up, patients with PAD alone were markedly less likely to receive disease-modifying pharmacotherapy, consisting of oral antiplatelet therapy; blood pressure control, preferably with ACE inhibitors; and lipid control with statins, relative to patients with coronary artery disease [10]. So far, limited data exist on the impact of guideline-directed pharmacotherapy on reintervention and survival rates after endovascular infrapopliteal revascularization, especially in CLI patients. One prior smaller study investigated 380 CLI patients who underwent diagnostic angiography or endovascular treatment regarding the benefit of statin therapy. Statins were prescribed for 246 patients. Statin therapy was associated with lower 1-year rates of MACCE (stroke, myocardial infarction or death) and a significantly better amputation-free survival and lesion patency (*p* < 0.05) in this study, but no longer follow-up was available. The effect of statin therapy on freedom from CD TLR was not investigated. Compared with our study cohort (73.7%), the rate of statin prescription was slightly lower at approximately 65% (11). However, a comparison with other prior studies of CLI patients shows that statin use is even significantly less frequent, ranging from 23% to 49% [15,16,17,18]. Aiello et al. [15] studied CLI patients undergoing endovascular treatment and reported 24-month outcomes showing improved primary and secondary vessel patency, limb salvage rates and overall survival with statin use. Multivariate regression analysis showed that statin therapy was also independently associated with improved limb salvage [15].

Interestingly, in the multivariate Cox regression analysis, we observed a significant reduction in mortality of about 33% with the use of beta-blockers. A possible explanation here is certainly the high proportion of patients with heart failure in our study cohort (see Table 1 and Table 3), where beta blocker therapy clearly improves prognosis and reduces mortality. These findings highlight the need to evaluate PAD and especially CLI patients for the presence of concomitant heart failure, as disease-modifying pharmacotherapy could substantially reduce the observed high mortality rates in this patient population. Although it has been postulated that beta-blocker therapy can potentially worsen limb perfusion, relevant comorbidities must be considered before discontinuation is contemplated.

## 6. Limitation

The limitations of the study include the sample size of this single-center, non-controlled study. As is well known with non-controlled studies, effect sizes tend to be overestimated. Long-term data would help to better differentiate between short- and long-term effects. All patients in this study cohort were initially classified as Fontaine stage IIb, in line with the local standard of care, and then reclassified for this data analysis according to the Rutherford clinical category, limiting the validity of this classification.

## 7. Conclusions

There is still an important underuse of OMT in patients undergoing infrapopliteal interventions, which is even more pronounced in CLI despite a signal for its benefit regarding all-cause mortality and major amputation-free survival. Further education and awareness of the benefits of optimal medical therapy for patients with IC and CLI are needed to increase prescription rates.

## Figures and Tables

**Table 1:** Patient characteristics.

	Total (N = 552)	IC (N = 145)	CLI (N = 407)	*p*-Value
Demographics				
Age	72.8 ± 11.0	71.2 ± 11.4	73.4 ± 10.8	0.03
Male gender	403 (73.0%)	106 (73.1%)	297 (73.0%)	0.98
BMI (kg/m^2^)	27.3 ± 5.1	27.2 ± 4.4	27.3 ± 5.3	0.98
BMI > 30 kg/m^2^	140 (25.4%)	35 (24.1%)	105 (25.9%)	0.68
Target limb characteristics				
Any prior limb intervention (ipsi- and/or contralateral)	392 (71.0%)	105 (72.4%)	287 (70.5%)	0.67
Endovascular	351 (63.6%)	102 (70.3%)	249 (61.2%)	0.05
Surgical	189 (34.2%)	34 (23.4%)	155 (38.1%)	0.001
Any prior target limb intervention	360 (65.2%)	96 (66.2%)	264 (64.9%)	0.77
Endovascular	322 (58.3%)	93 (64.1%)	229 (56.3%)	0.10
Surgical	137 (24.8%)	26 (17.9%)	111 (27.3%)	0.03
Rutherford Class (RC)				<0.001
2	41 (7.4%)	41 (28.3%)		
3	104 (18.8%)	104 (71.7%)		
4	86 (15.6%)		86 (21.1%)	
5	271 (49.1%)		271 (66.6%)	
6	50 (9.1%)		50 (12.3%)	
ABI	0.55 ± 0.34	0.63 ± 0.28	0.51 ± 0.36	<0.001
Discharge Medication				
Antiplatelets	527 (95.6%)	138 (95.2%)	389 (95.8%)	0.75
Anticoagulants	223 (40.5%)	56 (38.6%)	167 (41.1%)	0.60
Statins	419 (76.0%)	110 (75.9%)	309 (76.1%)	0.95
Other lipid-lowering drug	14 (2.5%)	5 (3.4%)	9 (2.2%)	0.42
ß-Blocker	336 (61.0%)	80 (55.2%)	256 (63.1%)	0.10
ACE-inhibitor or AT-2 antagonist	399 (72.4%)	118 (81.4%)	281 (69.2%)	0.01
Other antihypertensive medication	350 (63.5%)	73 (50.3%)	277 (68.2%)	< 0.001
Cardiovascular risk factors				
Hyperlipidemia	430 (77.9%)	114 (78.6%)	316 (77.6%)	0.92
History of hypertension	527 (95.5%)	137 (94.5%)	390 (95.8%)	0.35
Smoking				0.01
Never	272 (49.3%)	57 (39.3%)	215 (52.8%)	
Current	134 (24.3%)	44 (30.3%)	90 (22.1%)	
Prior	146 (26.4%)	44 (30.3%)	102 (25.1%)	
Diabetes	329 (59.6%)	63 (43.4%)	266 (65.4%)	< 0.001
Medical history				
Coronary artery disease	238 (43.1%)	55 (37.9%)	183 (45.0%)	0.10
Prior MI	89 (16.1%)	14 (9.7%)	75 (13.6%)	0.01
Heart failure	200 (36.2%)	36 (24.8%)	164 (40.3%)	0.001
Cerebrovascular disease	120 (21.7%)	21 (14.5%)	99/402 (24.3%)	0.01
Renal function				
Chronic renal insufficiency *	198 (35.9%)	45/145 (31.0%)	153/407 (37.6%)	0.11
Kidney failure **	39 (7.1%)	5 (3.4%)	34 (8.4%)	0.04
Pulmonary disease	99 (17.9%)	19 (13.1%)	80 (19.7%)	0.07

Continuous data are presented as means ± SD; categorical data are given as counts (percentage). IC = intermittent claudication; CLI = critical limb ischemia; BMI = body mass index; ABI = ankle brachial index; MI = myocardial infarction. ACE = Angiotensin-converting enzyme; AT-2 = Angiotensin-2. * Defined as estimated glomerular filtration rate <60 mL/min/1.73 m^2^ and ≥15 mL/min/1.73 m^2^. ** Defined as estimated glomerular filtration rate <15 mL/min/1.73 m^2^ or requirement of renal replacement therapy.

**Table 2:** Lesion and procedural characteristics.

	Total (N = 552)	IC (N = 145)	CLI (N = 407)	*p*-Value
Lesion characteristics				
Lesion length (mm)	208 ± 127	179 ± 130	218 ± 124	<0.001
Lesion type				0.52
De novo lesion	416 (75.4%)	112 (77.2%)	304 (74.7%)	
Restenotic lesion	111 (20.1%)	25 (17.2%)	86 (21.1%)	
In-stent restenosis	25 (4.5%)	8 (5.5%)	17 (4.2%)	
Severity of lesion				0.01
Stenosis	189 (34.2%)	62 (42.8%)	127 (31.2%)	
Chronic occlusion	363 (65.8%)	83 (57.2%)	280 (68.8%)	
Calcification				0.93
None/Mild	341 (61.8%)	90 (62.1%)	251 (61.7%)	
Moderate/Severe	211 (38.2%)	55 (37.9%)	156 (38.3%)	
Lesion location				
Tibioperoneal trunk	162 (29.3%)	59 (40.7%)	103 (25.3%)	<0.001
Anterior tibial artery	287 (52.0%)	57 (39.3%)	230 (56.5%)	<0.001
Posterior tibial artery	139 (25.2%)	33 (22.8%)	106 (26.0%)	0.43
Peroneal artery	143 (25.9%)	45 (31.0%)	98 (24.1%)	0.10
Procedural characteristics				
Retrograde puncture	82 (14.9%)	24 (16.6%)	58 (14.3%)	0.50
Simultaneous intervention of inflow vessels	229 (41.5%)	67 (46.2%)	162 (39.8%)	0.18
Simultaneous intervention of outflow vessels	57 (10.3%)	7 (4.8%)	50 (12.3%)	0.01
Atherectomy (directional or laser)	27 (4.9%)	9 (6.2%)	18 (4.4%)	0.39
Intraprocedural lysis	75 (13.6%)	26 (17.9%)	49 (12.0%)	0.08
Number of treated vessels				0.48
1	397 (71.9%)	101 (69.7%)	296 (72.7%)	
2–4	155 (28.1%)	44 (30.3%)	111 (27.3%)	
Infrapopliteal stent implantation	117 (21.2%)	40 (27.6%)	77 (18.9%)	0.03
Procedural success *	524 (94.9%)	139 (95.9%)	385 (94.6%)	0.55
Procedural complications	33 (6.0%)	7 (4.8%)	26 (6.4%)	0.50

Continuous data are presented as means ± SD; categorical data are given as counts (percentage). IC = intermittent claudication; CLI = critical limb ischemia. * Defined as residual stenosis < 50%.

**Table 3:** Key characteristics according to optimal medical therapy (OMT) at discharge.

	No OMT (N = 245)	OMT (N = 306)	*p*-Value
Demographics			
Age	72.9 ± 12.7	72.8 ± 9.4	0.93
Male gender	169 (69.0%)	233 (76.1%)	0.06
BMI (kg/m^2^)	26.4 ± 5.1	28.0 ± 5.0	<0.001
Target limb characteristics			
Any prior limb intervention (ipsi- and/or contralateral)	166 (67.8%)	225 (73.5%)	0.14
Prior target limb intervention	150 (61.2%)	209 (68.3%)	0.08
Endovascular	132 (53.9%)	189 (61.8%)	0.06
Surgical	64 (26.1%)	73 (23.9%)	0.54
Clinical status			0.07
IC	55 (22.4%)	90 (29.4%)	
CLI	190 (77.6%)	216 (70.6%)	
Cardiovascular risk factors			
Hyperlipidemia	164 (66.9%)	265 (86.6%)	<0.001
History of hypertension	223 (91.0%)	303 (99.0%)	<0.001
Smoking			0.02
Never	132 (53.9%)	139 (45.4%)	
Current	64 (26.1%)	72 (23.5%)	
Prior	50 (20.4%)	95 (31.1%)	
Diabetes	134 (54.7%)	194 (63.4%)	0.005
Medical history			
Coronary artery disease	90 (36.7%)	148 (48.4%)	0.01
Prior MI	32 (13.1%)	57 (18.6%)	0.06
Heart failure	90 (36.7%)	110 (35.9%)	0.76
Cerebrovascular disease	45 (18.4%)	73 (23.9%)	0.10
Renal function			
Chronic renal insufficiency *	82 (33.5%)	111 (36.3%)	0.49
Kidney failure **	31 (12.7%)	9 (2.9%)	<0.001
Lesion characteristics			
Lesion length (mm)	213.5 ± 123.7	203.4 ± 128.9	0.35
Lesion type			0.52
De novo lesion	187 (76.3%)	228 (74.5%)	
Restenotic lesion	45 (18.4%)	66 (21.6%)	
In-stent restenosis	13 (5.3%)	12 (3.9%)	
Severity of lesion			0.92
Stenosis	83 (33.9%)	105 (34.3%)	
Chronic occlusion	162 (66.1%)	201 (65.7%)	
Calcification			0.23
None/Mild	158 (64.5%)	182 (59.5%)	
Moderate/Severe	87 (35.5%)	124 (40.5%)	
Lesion location			
Tibioperoneal trunk	69 (28.2%)	92 (30.1%)	0.63
Anterior tibial artery	124 (50.6%)	163 (53.3%)	0.54
Posterior tibial artery	66 (26.9%)	73 (23.9%)	0.41
Peroneal artery	64 (26.1%)	78 (25.5%)	0.87
Procedural characteristics			
Simultaneous intervention of inflow vessels	102 (41.6%)	126 (41.2%)	0.91
Simultaneous intervention of outflow vessels	29 (11.8%)	28 (9.2%)	0.30
Atherectomy (directional or laser)	11 (4.5%)	16 (5.2%)	0.69
Intraprocedural lysis	38 (15.5%)	36 (11.8%)	0.20
Number of treated vessels			0.78
1	178 (72.7%)	219 (71.6%)	
2–4	67 (27.3%)	87 (28.4%)	
Infrapopliteal stent implantation	51 (20.8%)	66 (21.6%)	0.83
Procedural success ***	229 (93.5%)	294 (96.1%)	0.17
Procedural complications	14 (5.7%)	19 (6.2%)	0.81

Continuous data are presented as means ± SD; categorical data are given as counts (percentage). OMT = optimal medical therapy; BMI = body mass index; IC = intermittent claudication; CLI = critical limb ischemia; MI = myocardial infarction. * Defined as estimated glomerular filtration rate <60 mL/min/1.73 m^2^ and ≥15 mL/min/1.73 m^2^. ** Defined as estimated glomerular filtration rate <15 mL/min/1.73 m^2^ or requirement of renal replacement therapy. *** Defined as residual stenosis <50%.

## Data Availability

The data presented in this study are available on request from the corresponding author. The data are not publicly available due to data privacy.

## Abbreviations

ABI: Ankle-brachial index, ACE: Angiotensin-converting enzyme, BMI: Body mass index, CD TLR: Clinically driven target lesion revascularization, CHD: Coronary heart disease, CI: Confidence interval, CLI: Critical limb ischemia, DAPT: Dual antiplatelet therapy, HR: Hazard ratio, IC: Intermittent claudication, M: Mean, MI: Myocardial infarction, N: Number, OMT: Optimal medical therapy, PAD: Peripheral arterial disease, SD: Standard deviation.
